# Semi-Supervised Structural Prior-Guided Network for Space Target Component Segmentation in ISAR Images

**DOI:** 10.3390/s26154769

**Published:** 2026-07-27

**Authors:** Yonghua He, Aoxiang Pan, Yonggang Li, Jiahao Wang, Wei Qu, Weigang Zhu, Wenhang Ji

**Affiliations:** Space Engineering University, Beijing 101400, China; heyonghua1980@hgd.edu.cn (Y.H.); liyonggang@hgd.edu.cn (Y.L.); wjh_cwj@hgd.edu.cn (J.W.); quwei@hgd.edu.cn (W.Q.); zwg@hgd.edu.cn (W.Z.); jijiji0908@hgd.edu.cn (W.J.)

**Keywords:** semantic segmentation, semi-supervised learning, structural prior, Inverse Synthetic Aperture Radar (ISAR)

## Abstract

**Highlights:**

**What are the main findings?**

- The GMHC-ViT encoder broadens feature representation via multi-stream gating, effectively mitigating inter-class confusion, while the PGM injects shape and edge structural priors into weak-response channels, markedly improving boundary accuracy and component completeness.
- SSPNet consistently achieves superior segmentation performance across different annotation ratios and exhibits strong robustness under extremely limited labels and severe noise conditions.

**What are the implications of the main findings?**

- The proposed SSPNet enables highly accurate and label-efficient space target component segmentation, greatly reducing the reliance on expensive pixel-level annotations in ISAR interpretation tasks.
- The work demonstrates the benefit of explicitly encoding domain-specific structural knowledge into deep networks, offering a valuable reference for related tasks.

**Abstract:**

Segmenting key components of space targets using Inverse Synthetic Aperture Radar (ISAR) images is an important interpretation task in space situational awareness. However, the scarcity of pixel-level annotated data, inter-class confusion caused by morphological differences among multiple target classes, and the absence of structural priors for components restrict the performance improvement in existing deep models on this task. Therefore, this paper proposes a Semi-Supervised Structural Prior-Guided Network (SSPNet). First, a Gated Manifold-Constrained Hyper-Connections Vision Transformer (GMHC-ViT) encoder is proposed to broaden the feature representation space via parallel multi-feature streams with adaptive gating, thereby alleviating inter-class confusion and enhancing cross-category generalization. Second, a Prior-Guided Module (PGM) is proposed to extract shape and edge priors of components, and it adaptively enhances the weakly activated channels of encoder features through cross-attention, thereby injecting structural knowledge independent of image quality into the segmentation process. Furthermore, to effectively leverage large amounts of unlabeled data, a strong perturbation strategy tailored to the characteristics of ISAR images is designed for consistency regularization. Experimental results on a simulated ISAR dataset containing 38 classes of space targets demonstrate that SSPNet outperforms existing methods and exhibits strong segmentation capability even under low signal-to-noise ratio (SNR) conditions.

## 1. Introduction

With the vigorous development of global space exploration activities, the number of in-orbit spacecraft and space debris has rapidly increased, making high-precision space situational awareness capability crucial for ensuring the safety of space assets and implementing on-orbit servicing. Inverse Synthetic Aperture Radar (ISAR) [1], with its advantages of all-day and all-weather operation, long operating range, and high resolution, can provide two-dimensional high-resolution images of space targets, serving as an important means for structural interpretation and state assessment of space objects. Among these tasks, the accurate segmentation of key components of spacecraft not only enables precise estimation of component dimensions and identification of the main structure [2] but also provides information support for subsequent tasks such as attitude estimation [3,4] and three-dimensional reconstruction [5]. However, compared with natural scene images, ISAR images involve a complex imaging mechanism, making the interpretation of visual information highly challenging [6], and the high cost of ISAR image acquisition makes it difficult to obtain large-scale pixel-level annotated data [7]. These factors severely constrain the application of data-driven deep segmentation models in this field.

In recent years, although deep models based on CNN architectures [8] and Transformer architectures [9] have achieved excellent performance in optical image segmentation, their direct transfer to ISAR images often results in performance degradation [10,11]. This degradation stems from the differences in imaging mechanisms: ISAR images reflect the electromagnetic scattering distribution of targets rather than reflective properties, and are sensitive to changes in target attitude, leading to nonlinear geometric stretching and edge blurring in the images [12,13]. Moreover, existing semantic segmentation methods for space targets in ISAR images generally overlook two factors that are crucial to this task. First, the diversity of target categories is insufficient: most existing works are validated on only a few classes of space targets, typically no more than 10 classes [14,15,16]. Such small-scale settings fail to reflect the high diversity of target models in practical applications. Different classes of targets may exhibit significant differences in overall configuration, scale ratio, and the spatial distribution of scattering centers. An increase in the number of categories not only introduces the risk of inter-class shape confusion but also poses a significant generalizability challenge for models trained on a limited number of categories. Second, there is a lack of utilization of structural priors of space targets: previous methods generally neglect the strong structural priors inherent to space targets as man-made structures [14,15,16]. For many space targets, solar panels are often narrow and rectangular, the main body is typically polyhedral, and antenna reflectors feature parabolic contours [17], with the connections between various components following deterministic engineering logic. Such structural prior information is independent of image quality and can provide constraints for blurred edges or incomplete scattering regions. However, existing methods fail to embed prior knowledge about component geometry or spatial relationships into the learning process, leaving the entire decision-making process blind to component structure and thus limiting component segmentation accuracy.

In addition to the aforementioned limitations at the model level, the scarcity of pixel-level annotations further restricts the feasibility of fully supervised training in this field. On the one hand, the ISAR data available for training is relatively scarce compared with other domains; on the other hand, the annotation process heavily relies on the domain expertise of researchers in related areas. To leverage large amounts of unlabeled ISAR data with limited annotations, the semi-supervised learning paradigm has gradually gained attention. Currently, the widely adopted consistency regularization framework based on data perturbation has achieved remarkable success in optical image segmentation [18,19]. Its core idea is to apply perturbations of varying strengths to unlabeled images, forcing the model to produce consistent predictions and thereby providing additional supervisory signals. However, the perturbation strategies in such methods are designed for natural optical images, such as color jittering and random occlusion, with the implicit assumption that image semantics remain invariant under such pixel-value and spatial perturbations. For ISAR images, semantic information is carried by the relative intensity relationships among scattering points, and the above perturbations may destroy this critical information, thus impairing the benefit gained from unlabeled data.

To address the above issues, this paper proposes a Semi-Supervised Prior-Guided Network (SSPNet), a space target component segmentation model based on semi-supervised learning and structural prior guidance, which is jointly designed at both the network architecture and training strategy levels. In terms of architecture design, the model adopts an encoder–decoder architecture. The encoder employs a Gated Manifold-Constrained Hyper-Connections Vision Transformer (GMHC-ViT), which broadens the feature representation space by introducing multiple parallel feature streams, where the mixing among feature streams is dynamically performed by data-driven hyper-connection weights, and a gating mechanism is applied to the fusion results for selective enhancement, thereby effectively overcoming the insufficient generalizability caused by target diversity. The feature maps output by the encoder then pass through a lightweight Channel Scoring Module (CSM), which retains only the top-K channels with the highest scores; the remaining weakly activated channels are reconstructed with the aid of structural prior features. Through the Prior-Guided Module (PGM), shape features are extracted from the two-dimensional projection maps of typical space target components, and contour features from their edge maps. After fusion, these form a structural prior that encodes both geometric morphology and boundary information. This prior is then interactively fused with the weakly activated channels via cross-attention, explicitly injecting structural knowledge of components into the encoded features, thereby enhancing segmentation region localization and edge integrity. Since the encoded features fused with prior features are semantically rich enough, the decoder consists merely of several simple upsampling layers. In terms of training strategy, to cope with label scarcity, this paper adopts a semi-supervised framework based on consistency regularization and makes adaptive modifications to the strong perturbation strategy according to the characteristics of ISAR images, such as low-pass filtering and multiplicative speckle noise, thereby ensuring that unlabeled images maintain consistent component semantics after perturbation.

In summary, the main contributions of this paper are as follows.

- We propose SSPNet, which utilizes the GMHC-ViT encoder to broaden the feature space, enhances structural semantics through channel filtering and prior injection, and then generates segmentation masks via an upsampling decoder. During training, a strong perturbation strategy tailored to ISAR image characteristics is adopted in place of conventional perturbations to enable effective utilization of unlabeled data.
- We propose the GMHC-ViT encoder, which introduces manifold-constrained hyper-connections and a gating mechanism into the ViT, establishes parallel feature streams between layers, and employs data-dependent mixing weights and gating for selective enhancement. This design effectively broadens the feature representation space without substantially increasing the number of parameters, thereby improving multi-category generalization capability.
- We propose the PGM, which leverages the shape and edge information of typical space target components as structural priors, reshapes features of low-scoring channels via cross-attention, and explicitly injects component structural knowledge into the encoded features, thereby enhancing the boundary accuracy and structural integrity of the segmented regions.
- We construct an ISAR image segmentation dataset comprising 38 different classes of space targets, and comprehensively evaluate model performance under varying labeling ratios and signal-to-noise ratios (SNRs). Experimental results validate the effectiveness of the proposed method.

The remainder of this paper is organized as follows. Section 2 briefly reviews related work. Section 3 elaborates on the proposed SSPNet in detail. Section 4 presents comprehensive experimental results and analysis to demonstrate the effectiveness of the method. Finally, Section 5 draws the conclusions of this paper.

## 2. Related Work

### 2.1. Semantic Segmentation of Space Target ISAR Images

Early research on ISAR image segmentation primarily relied on traditional image processing techniques, such as methods based on intensity thresholding [20], edge extraction [21], region growing and clustering [22,23], and statistical models [24,25]. These methods typically require manual parameter tuning for specific targets and exhibit limited generalization capability.

With the rise of deep learning, researchers have begun transferring general semantic segmentation architectures to space target ISAR images and making adaptive modifications. For example, Zhu et al. [11] combined U-Net with a Siamese network, which first obtains a target mask through binary segmentation and then uses mask similarity matching to achieve component label prediction. Kou et al. [16] designed CL-NL-Unet, which introduces a non-local attention mechanism to capture the global symmetric structure of targets and leverages contrastive learning to enhance segmentation of small components. Li et al. [10] constructed the multi-modal scattering feature perception framework SPSF, which adaptively fuses texture features of ISAR images with quantitative scattering information from complex-valued echo data, thereby improving segmentation robustness under low SNR conditions. Du et al. [15] designed DADMC-U2Net, which employs multi-label semantic segmentation and dense multiplicative connections to reconstruct the complete two-dimensional projection contours of components and optimizes boundary accuracy of overlapping regions through a fusion loss function. Zhong et al. [14] proposed the scattering characteristic guided network SCGN, which uses explicit supervision to learn the scattering center distributions of different components and utilizes this as auxiliary information to guide the segmentation head, achieving coarse-to-fine segmentation of components. To address the scarcity of training data, Heslinga et al. [7] trained a baseline segmentation model on a large number of low-fidelity simulated ISAR images and combined it with few-shot domain adaptation techniques, thereby significantly improving segmentation performance when only a small number of high-fidelity simulated images are available. Yang et al. [26] proposed the joint category-pose guidance network CPGNet, which adopts a fine-grained categorical training paradigm and integrates target category and pose information into semantic features through a category-pose guidance module, thereby alleviating class confusion in multi-target component recognition. In addition, Duan et al. [27] started from differences in weak textural features within pixel neighborhoods and achieved pixel-level component segmentation by training a patch-based classification network, demonstrating good generalization ability under few-shot settings.

These deep learning methods have advanced space target component segmentation from multiple dimensions, including network architecture design, scattering feature utilization, and training strategies. Although some works [10,14] introduce scattering characteristic information into the segmentation process, this information is extracted from the input data itself, is highly dependent on the quality of the input data, and is prone to inter-class bias. In contrast, the structural prior adopted in this paper is independent of input data quality and is more intuitive.

### 2.2. Structure Prior Guided Image Segmentation

Structural priors refer to prior knowledge concerning the geometric morphology, topological relationships, boundary contours, and spatial distribution of target objects. Introducing such priors into deep learning models can effectively compensate for the limitations of purely data-driven methods in scenarios involving low contrast, blurred boundaries, and significant shape variation in targets [28,29,30]. In recent years, how to effectively embed explicit structural knowledge into deep neural networks has become an important research direction for improving the robustness and generalization of segmentation.

Structural priors have been widely utilized to guide segmentation in fields such as medical imaging, industrial vision, and remote sensing image analysis. For instance, in medical image segmentation, Wyburd et al. [31] proposed TEDS-Net, which encodes the topological features of anatomical structures as topology-preserving deformation fields to deform an initial shape prior with correct topology onto the target image, thereby ensuring the topological correctness of segmentation results. Zhao et al. [32] designed ESVC-Net, an edge-enhanced semi-supervised vertical convolutional network for tubular structures, which leverages an auxiliary edge detection task to learn edge-related features of tubular structures and integrates them into the segmentation decoder via an adaptive enhancement module to enhance boundary awareness. Zhang et al. [33] proposed the structural prior-guided network SPG-Net, which propagates structural information from high-level features to low-level features through a local affinity feature fusion module, and jointly supervises training at both the pixel and structure levels using a multi-scale structural similarity loss, effectively alleviating the problem of discontinuous boundary segmentation of corneal endothelial cells. In industrial vision, He et al. [34] proposed a prior-aware weakly supervised segmentation model for automobile body surface defects and designed a boundary-guided prior constraint loss that uses the spatial constraints embedded in bounding box annotations to guide mask generation, thereby achieving fine defect segmentation with only box-level annotations. Sun et al. [35] proposed DEPANet, a differentiable edge-guided pyramid aggregation network for strip steel surface defects, which extracts multi-scale edge information through learnable Laplacian operators and adaptively fuses edge priors with global features of the backbone via an edge-aware feature aggregation module, significantly improving the segmentation accuracy of defect boundaries. In remote sensing image segmentation, Wang et al. [36] proposed ReSMamba, a relation-structure-aware Mamba segmentation network that embeds topological prior information into the state space and designs a directional connectivity prediction module to explicitly model multi-directional spatial connectivity relationships between pixels, thereby enhancing boundary continuity and small target segmentation capability. Li et al. [37] proposed GPINet, a geometry-prior-guided interactive network that fuses CNN and Transformer features through a dual-branch local-global interaction module and iteratively updates geometry priors via a geometry prior generation module to guide the decoder in feature recovery, thus improving the edge accuracy of ground objects in remote sensing images. Xie et al. [38] integrated a learnable morphological skeleton prior into the SAM decoding process in the form of a variational model, constraining the segmentation output through a smoothed morphological skeleton operator to preserve the fine structures and topological connectivity of objects.

These works have validated, within their respective domains, the effectiveness of structural priors in improving segmentation accuracy. However, research on segmentation methods guided by such structural priors in the domain of space target ISAR images remains relatively scarce. Considering that space targets possess highly standardized structural characteristics, introducing such prior information into segmentation models holds promise for further enhancing segmentation accuracy.

### 2.3. Semi-Supervised Segmentation Based on Perturbation Consistency

While weakly supervised and few-shot segmentation [39,40] offer alternative solutions for label efficiency, this paper focuses on semi-supervised consistency regularization. Semi-supervised learning aims to train models jointly with a small number of labeled samples and a large number of unlabeled samples, thereby mitigating the scarcity of pixel-level annotations [18]. Consistency regularization methods represent a major category of semi-supervised paradigms, whose core idea is to apply different forms of perturbations to the same unlabeled image to generate multiple views, and then enforce consistency in the model’s predictions across these views [41]. This consistency-based mechanism converts unlabeled data into effective supervisory signals, thus improving model performance in low-annotation settings [42].

Current mainstream semi-supervised methods generally employ carefully designed perturbation strategies to construct consistency constraints. For example, Mean Teacher [43] is an early representative semi-supervised framework that builds a teacher model via exponential moving average (EMA) and constrains the student model to produce predictions consistent with the teacher’s predictions on either noisy or clean inputs, and has been widely adopted in tasks such as medical image segmentation [44]. Building on this, FixMatch [45] pushes consistency learning to a new level: it uses a weakly perturbed view to generate high-confidence pseudo-labels, and then enforces consistency between a strongly perturbed view and those pseudo-labels, thereby significantly boosting semi-supervised learning performance. UniMatch [46] further expands the perturbation space, applying not only intensity perturbations to images but also channel-level Dropout in the feature space, thereby jointly enhancing the robustness of consistency at both the image and feature levels. In addition, Cross Pseudo Supervision (CPS) [47] approaches the problem from a network architecture perspective, in which two subnetworks with identical structures but different initializations mutually generate pseudo-labels and impose cross-supervision on each other, thereby using the network’s own discrepancy as an implicit perturbation source to avoid over-reliance on manually designed strong perturbations. These methods have achieved excellent results on natural optical images, thereby collectively demonstrating the effectiveness of the “perturbation consistency” paradigm. However, due to certain differences between ISAR imaging mechanisms and optical imaging mechanisms, specialized perturbation strategies need to be designed.

## 3. Materials and Methods

This section details the architecture of the proposed SSPNet. The model integrates semi-supervised learning with structural prior guidance, broadens the feature representation space through the GMHC-ViT, and injects geometric and boundary knowledge of space target components into the segmentation network via the PGM, thereby achieving accurate component segmentation for multiple classes of space targets in ISAR images under scarce-annotation conditions.

### 3.1. Architecture Overview

In recent years, deep neural networks have shown significant potential in semantic segmentation tasks, providing a novel paradigm for the automatic interpretation of space target ISAR images. Although related studies have improved models from multiple perspectives, component-level semantic segmentation of space target ISAR images still faces three limitations: the scarcity of pixel-level annotations, the absence of structural prior knowledge of components, and insufficient model generalization across multiple target categories. These factors collectively constrain the development of component segmentation for space targets.

To address the above challenges, SSPNet combines a prior-guided network architecture with a semi-supervised training strategy tailored to ISAR image characteristics. Its core idea is as follows: first, broadening the feature representation space to handle diverse target types; then, exploiting shape and edge priors obtained from two-dimensional projection maps of typical components to selectively enhance weakly activated channels; and finally, decoding the enhanced features to generate segmentation masks. In terms of training, a strong perturbation strategy tailored to the characteristics of ISAR images is designed to generate effective pseudo-supervisory signals. This design enables SSPNet to achieve favorable segmentation performance across multiple classes of space targets even when only a small amount of data is annotated.

**Figure 1:**
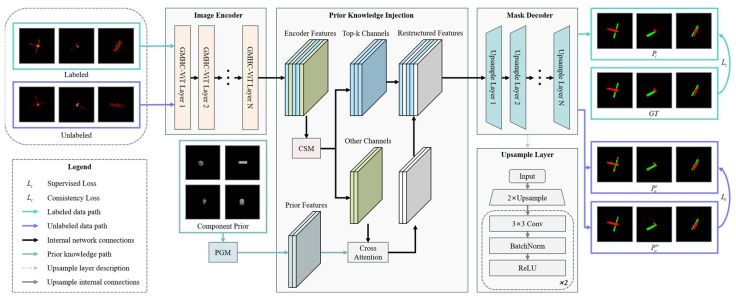
Overall structure of SSPNet. The framework consists of four main stages: multi-stream feature encoding via the GMHC-ViT image encoder, structural prior knowledge injection through the PGM, mask resolution recovery using a lightweight decoder, and semi-supervised training based on a consistency regularization loss.

The overall architecture of SSPNet is illustrated in Figure 1. The input ISAR image first passes through the GMHC-ViT encoder. This encoder broadens the feature space by maintaining multiple parallel feature streams coupled with a gating mechanism, thereby better adapting to scattering variations among multi-class targets. The encoded feature maps are then processed by the CSM, which ranks the channels according to their information content, retaining only the top-K channels with the highest scores, while the remaining low-scoring channels are regarded as candidates for injecting prior knowledge. In the PGM, the structural prior consists of shape and edge features of components; after fusion, these two features interact with the low-scoring channels via a cross-attention mechanism to enhance the feature representations of the low-scoring channels. The reconstructed channels are merged with the retained top-K channels and fed into the decoder. Since the encoded features are semantically rich and the shallow features do not contain any prior information, the decoder is simply composed of stacked upsampling layers without skip connections. Ultimately, high-resolution segmentation masks of space targets are generated. In the semi-supervised training phase, an improved strong perturbation strategy is adopted for unlabeled ISAR images, in which a consistency regularization loss is imposed on the predictions from a weakly perturbed view and two different strongly perturbed views of the same unlabeled image, thereby improving the supervisory effect of unlabeled data.

### 3.2. Gated Manifold-Constrained Hyper-Connections Vision Transformer (GMHC-ViT)

The standard ViT [48] propagates information through a single feature stream layer by layer. Although it is capable of capturing global context through the self-attention mechanism, its fixed-width feature representation struggles to simultaneously accommodate the disparities in scattering and geometric characteristics across multiple classes of space targets. The differences in the distribution and morphology of scattering centers among different target classes make the single feature stream prone to developing inter-class feature bias when confronted with such diversity, thereby limiting cross-category generalization capability.

Hyper-Connections (HC) [49] broaden the feature representation space at the architectural level by extending the standard residual connections to multiple parallel feature streams, and have demonstrated significant performance improvements in the pre-training of large language models. However, the unconstrained residual mapping matrices of HC disrupt the identity mapping property, leading to training instability. Recently, DeepSeek proposed Manifold-Constrained Hyper-Connections (mHC) [50], which constrain the residual mapping matrices to be doubly stochastic matrices, restoring training stability while inheriting the performance advantages of multiple feature streams of HC.

Inspired by this, this paper introduces mHC into the field of semantic segmentation. On this basis, to further enhance the quality of multi-stream feature fusion, this paper introduces a gating mechanism. Specifically, the gating is placed after the inter-stream mixing and the main module output fusion: after multiple parallel feature streams are recombined, they are added, according to learnable weights, to the main module output processed by the self-attention or feedforward module, and subsequently a Sigmoid gating is applied to the fusion result for element-wise selective enhancement. The primary role of this gating is to adaptively regulate the proportion of effective information in each component of the fused features, wherein discriminative features relevant to the current target’s scattering pattern are enhanced, while noise or irrelevant components introduced during cross-stream mixing are suppressed. Through this selective information regulation, the multi-stream features can more accurately adapt to the scattering and geometric characteristics of different space targets, thereby improving the feature quality and generalization capability of the encoder in multi-category scenarios.

**Figure 2:**
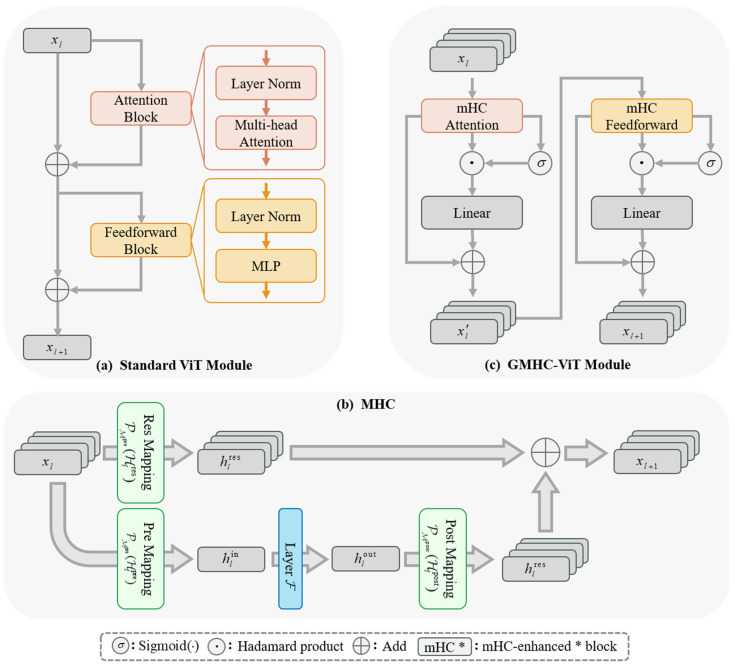
(**a**) Standard ViT module [48], which mainly includes a self-attention submodule and a feedforward submodule. (**b**) Standard mHC [50], which extends the residual connection and uses a special design to ensure training stability. (**c**) Proposed GMHC-ViT module, which employs mHC to modify the standard ViT module and incorporates a gating mechanism to modulate information flow.

The composition of the GMHC-ViT module is illustrated in Figure 2. A standard ViT module can be divided into two submodules according to the residual connection: the self-attention submodule and the feedforward submodule. These two submodules are both restructured using the mHC computation method, and a gating mechanism is added to each, thereby ensuring that both self-attention and feature transformation benefit from the gated multi-stream representation. The computation process is as follows. Let $X_{mHC}$ be the output after mHC completes inter-stream mixing and main module fusion, and then the gating path can be expressed as:

(1) $$X_{out}=\mathrm{Linear}(X_{mHC}⊙\sigma (X_{mHC}))+X_{mHC}$$

where σ(⋅) denotes the Sigmoid function, ⊙ represents the Hadamard product, and Linear(⋅) denotes a learnable linear transformation with matching input and output dimensions.

The GMHC-ViT encoder is constructed by stacking multiple aforementioned modules. The initial feature streams of the first module are obtained by replicating and expanding the input single-stream tokens, where is the number of feature streams and D is the token dimension. For subsequent modules, the input feature streams are the output feature streams of the preceding module. Finally, the multi-stream features are averaged along the stream dimension to fuse back into a single-stream representation. Benefiting from the synergistic design of multi-stream gating, GMHC-ViT effectively broadens the feature representation space without substantially increasing inference overhead, thereby providing a robust feature foundation for subsequent modules.

### 3.3. Component Prior Knowledge Injection

To introduce the structural priors of space targets into the segmentation network, this paper designs a structural prior injection mechanism following the image encoder, comprising the CSM and the PGM. The CSM first evaluates the channels of the encoded features based on their information content and filters them, thereby identifying weakly activated channels as candidates for enhancement. The PGM subsequently extracts the structural prior from the two-dimensional projection maps and edge maps of target components, and reconstructs the weakly activated channel features via cross-attention, so that the encoded features carry geometric and boundary knowledge of components before being fed into the decoder. The specific designs of the two modules are elaborated below.

#### 3.3.1. Channel Scoring Module (CSM)

The feature maps produced by the image encoder contain multiple channels, but not all channels contribute equally to the segmentation task. Some channels carry clear discriminative information for components, while others exhibit weak responses and ambiguous semantics. Directly feeding these weak channels into the decoder not only introduces redundant information but may also compromise boundary localization and reduce the decoder’s ability to focus on salient semantics. Therefore, a lightweight channel filtering mechanism is required to evaluate the information importance of each channel.

**Figure 3:**
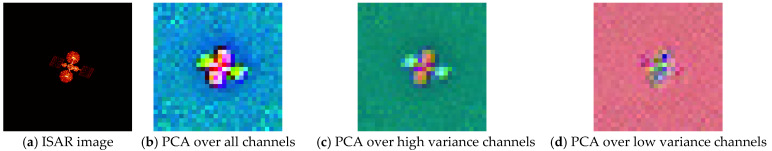
PCA dimensionality reduction results of encoded features. (**a**) is the input ISAR image; (**b**) shows the result after PCA dimensionality reduction on all channels of the encoded features; (**c**) shows the result after dimensionality reduction on high variance channels (top 100), with a variance proportion of 84.76%; (**d**) shows the result after dimensionality reduction on low variance channels (bottom 100), with a variance proportion of 6.71%.

To visually demonstrate the differences in the importance of information carried by different channels, this paper adopts Principal Component Analysis (PCA) to perform channel-wise visualization of the encoder output features. Specifically, the feature maps are flattened across spatial dimensions and then reduced to three principal components via PCA, thereby generating pseudo-color visualization images. Figure 3 shows the dimensionality reduction results under different numbers of channels. It can be observed that when the channels are ranked according to their spatial variance, the PCA reduction results of different channels exhibit noticeable discrepancies: the activations of high-variance channels are mainly concentrated in the target component regions, containing a high amount of information; whereas low-variance channels show lower response intensity in the component regions and manifest more as random fluctuations, containing a low amount of information. The above phenomenon indicates that different feature channels carry varying amounts of information and thus need to be treated differently. Although spatial variance serves as an intuitive metric, it is not suitable as a criterion for channel selection.

**Figure 4:**
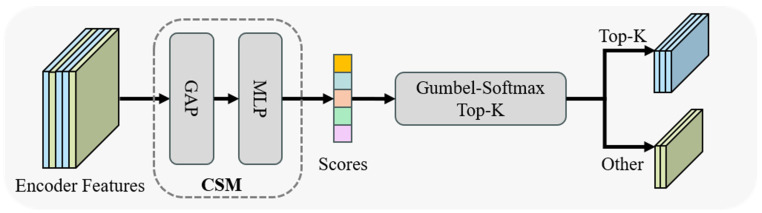
Lightweight CSM. The CSM computes a score for each channel of the encoded feature maps and selects strong and weak channels according to the scores.

Based on the above observations, this paper designs the CSM, which adaptively quantifies the information importance of each channel and accordingly divides them into strong and weak channels. To avoid introducing excessive parameters or computational overhead, the CSM is designed to be sufficiently lightweight. The structure of the CSM is shown in Figure 4, whose core is a learnable scoring network comprising global average pooling and an MLP.

(2) $$Scores=\mathrm{MLP}(\mathrm{GAP}(F_{enc}))$$

where $F_{enc}\in {\mathbb{R}}^{C\times H\times W}$ denotes the encoder features, and GAP(⋅) represents global average pooling over the spatial dimensions, compressing the features into a channel descriptor vector $z\in {\mathbb{R}}^{C}$.

Based on the scoring results, the CSM divides the feature channels into two groups: the top-K channels with the highest scores are retained as strong information channels $F_{strong}\in {\mathbb{R}}^{K\times H\times W}$ and directly fed into the subsequent decoder; the remaining C−K channels are marked as weakly activated channels $F_{weak}\in {\mathbb{R}}^{(C-K)\times H\times W}$ and serve as candidates for enhancement by the Prior Guided Module. Notably, during channel selection, the Gumbel-Softmax [51] trick is adopted, thereby enabling the conversion of the non-differentiable discrete Top-K selection into a continuous approximation operation that enables end-to-end training.

#### 3.3.2. Prior-Guided Module (PGM)

After the CSM divides the encoded features into strong information channels and weak information channels, a direct approach would be to simply discard the weak channels and feed only the strong channels into the decoder. However, although the response intensity of weak channels is low, their activation regions often exhibit spatial correspondence with component structures, merely lacking sufficient discriminative semantics. Simply zeroing them out would discard these potential spatial cues, and such a loss may lead to degraded segmentation integrity. On the other hand, the scattering characteristics of ISAR images inherently result in component boundaries that are often discontinuous and of low contrast; relying solely on the features extracted from images by the encoder cannot guarantee the accuracy of segmentation boundaries and the structural integrity of components. By contrast, the structural priors of space targets are independent of ISAR image quality and can provide support for improving segmentation accuracy. Therefore, a key question is how to construct suitable structural priors and inject them into the encoded features to alleviate the semantic deficiency of weakly activated channels.

In response to the above issue, this paper designs the PGM. Its core idea is to utilize CAD models of typical space target components to render two-dimensional projection maps, from which overall shape features and edge features are extracted and fused into a unified structural prior representation. The shape prior provides global geometric constraints for the components, while the edge prior provides spatial localization of local contours; the two complement each other and jointly form a complete structural description of the components. Subsequently, through a cross-attention mechanism, this prior representation is used as a guiding signal to perform feature enhancement on the weakly activated channels selected by the CSM—with the weak channel features serving as Query, and the fused prior features serving as Key and Value, thereby enabling the weak channel features at each spatial position to be adaptively reshaped based on their semantic relevance to the prior feature regions. The enhanced weakly activated channels are merged with the originally retained strong information channels in their original order, forming complete encoded features that contain both data-driven scattering semantics and injected structural prior knowledge of the components.

**Figure 5:**
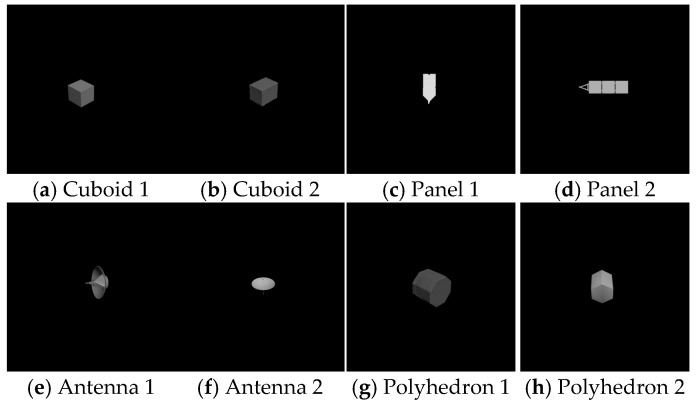
Rendered 2D CAD projections of typical spacecraft components, which illustrate four representative types (cuboid, panel, antenna, polyhedron). For each type, two shape variants are provided to reflect the intra-category shape diversity commonly observed across different space targets.

Although CAD-rendered projection maps differ from ISAR images in formation mechanism, the geometric configuration, relative positions, and spatial topology of the target components reflected by both are consistent, thereby providing geometric and topological constraints independent of image quality for ISAR segmentation. The two-dimensional projection maps of typical components are shown in Figure 5. This multi-type prior design enables the Prior-Guided Module to be exposed to a wider range of component geometric patterns during training, thereby prompting it to learn structure-adaptive prior representations, so that it can still provide effective structural guidance when facing different configurations of various types of space target components.

**Figure 6:**
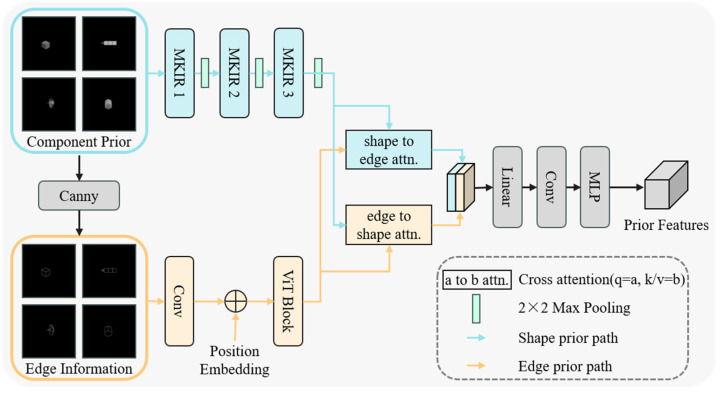
Architecture of the PGM, depicting the extraction process of structural prior features for enhancing the weakly activated channels selected by the CSM.

The architecture of the PGM is shown in Figure 6, depicting only the construction process of the structural prior features. The extraction of the structural prior consists of three parts: a shape feature extraction branch, an edge feature extraction branch, and bidirectional cross-attention fusion.

(1) Shape feature extraction: The shape branch processes the CAD-rendered two-dimensional projection shape map, which contains dense texture information and hierarchical geometric information. To this end, this branch employs three cascaded MKIR [52] blocks for feature extraction, where 2 × 2 max pooling is applied between blocks for progressive downsampling. Each MKIR block performs depthwise separable convolutions with three kernel sizes—1 × 1, 3 × 3, and 5 × 5—in parallel within the same layer, thereby enabling the network to simultaneously capture spatial patterns at different receptive fields and obtain rich multi-scale shape features while remaining lightweight. The mathematical formulation is as follows.

(3) $$\begin{array}{l} F_{\mathrm{shape}}^{\left(0\right)}=I_{\mathrm{shape}}\\ F_{\mathrm{shape}}^{\left(l\right)}=\mathrm{MaxPooling}\left({\mathrm{MKIR}}^{\left(l\right)}\left(F_{\mathrm{shape}}^{\left(l-1\right)}\right)\right),\;\;\;\;l=1,2,3 \end{array}$$

where $I_{\mathrm{shape}}$ denotes the input shape prior, and $F_{\mathrm{shape}}$ denotes the shape features.

(2) Edge feature extraction: The shape map is processed by Canny edge detection to obtain an edge map, which possesses binarized sparse contour information. The semantics of contours heavily depend on their spatial positions, which requires the network to have the capability of modeling long-range dependencies among discrete contour segments. Therefore, the edge branch adopts a Vision Transformer (ViT) module, establishing long-range topological relationships among contour segments via positional encoding and global attention. This design effectively compensates for the shape branch’s deficiency in perceiving boundary details. The mathematical formulation is as follows.

(4) $$F_{\mathrm{edge}}=\mathrm{ViT}\text{\_}\mathrm{Block}(\mathrm{Conv}(I_{\mathrm{edge}})+E_{\mathrm{pos}})$$

where $I_{\mathrm{edge}}$ denotes the input edge prior, $F_{\mathrm{edge}}$ denotes the edge features, and $E_{\mathrm{pos}}$ denotes the position embedding.

(3) Bidirectional cross-attention fusion: Shape features are adept at describing the global morphology of components but lack precise boundary localization, while edge features excel at depicting contour orientation but lack holistic geometric semantics. Therefore, after obtaining the shape and edge features, the PGM performs bidirectional cross-attention to fuse these two complementary structural priors. Specifically, using shape features as Query and edge features as Key and Value produces edge-enhanced shape features, while edge features used as Query and shape features as Key and Value produce shape-enhanced edge features. The two enhanced features are concatenated and fused through a fully connected layer, and then mapped via a convolutional layer and an MLP to form the final structural prior features, which are subsequently used to enhance the weakly activated channels from the CSM. The mathematical formulation is as follows.

(5) $$\begin{array}{l} F_{se}=\mathrm{CrossAttn}(F_{\mathrm{shape}},F_{\mathrm{edge}}),\\ F_{es}=\mathrm{CrossAttn}(F_{\mathrm{edge}},F_{\mathrm{shape}}),\\ F_{\mathrm{prior}}=\mathrm{MLP}\left(\mathrm{Conv}\left(\mathrm{Linear}\left(\mathrm{Contact}[F_{se},F_{es}]\right)\right)\right) \end{array}$$

where $F_{\mathrm{prior}}$ denotes the extracted prior features.

### 3.4. Strong Perturbation Strategy and Loss Functions

To effectively leverage a large number of unlabeled ISAR images with limited annotations, this paper adopts a semi-supervised training framework based on consistency regularization. However, the perturbation strategies in traditional semi-supervised methods are designed for optical images, whereas ISAR image semantics are determined by scattering center intensities and their relative spatial distributions; this discrepancy renders optical perturbations destructive to the physical semantics of components.

To address this issue, this paper employs a strong perturbation strategy tailored to ISAR characteristics, comprising the following three perturbation operators. Each time the strategy is applied to an unlabeled image, one or more operators are randomly selected from the operator pool and combined in series, where the key parameters of each operator are randomly sampled within a preset range.

Multiplicative speckle noise is used to simulate intensity fluctuations caused by coherent speckle. In this paper, multiplicative noise based on the Rayleigh distribution is adopted.

(6) $$I_{\mathrm{sn}}(x,y)=I(x,y)\cdot n(x,y),\;\;\;\;n(x,y)\sim \mathrm{Rayleigh}(\sigma )$$

where I denotes the input image and $I_{\mathrm{sn}}$ denotes the noisy image. The probability density function of the Rayleigh distribution is $p(n)=\frac{n}{\sigma^{2}}\exp\left(-\frac{n^{2}}{2\sigma^{2}}\right)$, n≥0, with the scale parameter σ∈[0.5,1.5] controlling the noise intensity.

Low-pass filtering is adopted, which truncates high-frequency components in the frequency domain to simulate detail loss caused by limited resolution.

(7) $$I_{\mathrm{lp}}=F^{-1}\left\{F\{I\}\cdot M_{r}(U,V)\right\},\;\;\;\;M_{r}(U,V)=\left\{\begin{array}{l} 1,\;\;\;\;\sqrt{U^{2}+V^{2}}\leq r\\ 0,\;\;\;\;\mathrm{Otherwise} \end{array}\right.$$

where $I_{\mathrm{lp}}$ represents the image after adding noise, and F and $F^{-1}$ denote the two-dimensional Fourier transform and inverse Fourier transform, respectively; (U,V) are the frequency-domain coordinates; the cutoff frequency is $r=f_{c}\cdot \min(H,W)$, where $f_{c}$ is the proportional coefficient; H and W are the height and width of the image.

Frequency-domain phase error blurring is used to simulate structural blurring and detail degradation induced by phase errors.

(8) $$I_{\mathrm{pc}}=\left|F^{-1}\left\{F\{I\}\cdot \exp(j\alpha (U^{2}+V^{2}))\right\}\right|$$

where $I_{\mathrm{pc}}$ denotes the blurred image, and α∈[30,100] controls the intensity of the phase error.

The total training loss of the model consists of the weighted sum of the supervised loss on labeled data and the consistency loss on unlabeled data, both of which are computed using cross-entropy loss:

(9) $$L=\lambda \cdot L_{s}+\frac{1-\lambda }{2}\cdot (L_{c}^{\left(1\right)}+L_{c}^{\left(2\right)})$$

where $L_{s}$ denotes the supervised loss for labeled data, and $L_{c}^{\left(1\right)}$ and $L_{c}^{\left(2\right)}$ denote the consistency losses corresponding to the two strongly perturbed views of unlabeled data, respectively. λ is a weight coefficient that is adjusted according to the training stage: it takes a larger value during the early training phase to primarily establish basic segmentation capability using labeled data, and gradually decreases as training progresses to increase the contribution ratio of the unlabeled loss.

## 4. Results and Discussion

This paper designs a series of experiments to demonstrate the effectiveness of the proposed method. Section 4.1 introduces the ISAR dataset used in the experiments; Section 4.2 specifies the key parameter configurations and evaluation metrics; Section 4.3 presents comparative experiments between the proposed method and representative algorithms of the same type; based on this, an efficiency comparison is provided in Section 4.4; ablation studies are conducted in Section 4.5 to deeply analyze and verify the effectiveness of each key module; and Section 4.6 presents experiments under different SNR conditions.

### 4.1. Datasets

**Figure 7:**
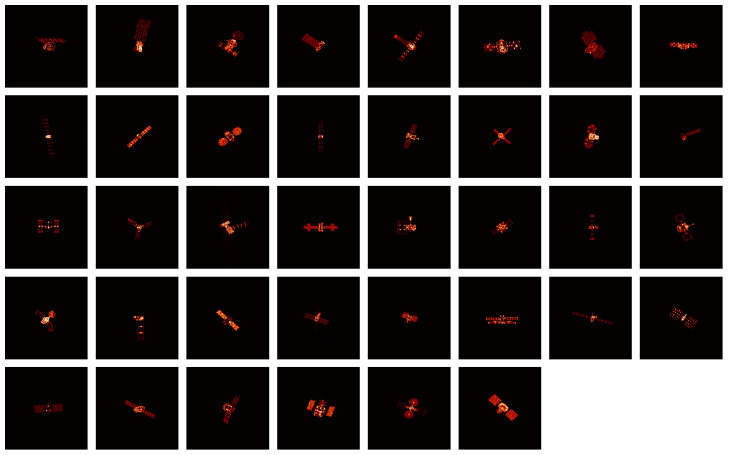
ISAR images of 38 space targets, arranged from left to right and top to bottom: ST1 to ST38.

A dataset is constructed using simulated ISAR images of 38 different classes of space targets. Each class is imaged under multiple attitudes, as shown in Figure 7. The simulation parameters are set as follows: center frequency of 17 GHz, bandwidth of 2 GHz, ISAR image resolution of 0.075 m, and image dimensions of 512 × 512 pixels. The modeling of the radar and target is based on the turntable model, and after radar echoes are obtained, the Range-Doppler algorithm is used for ISAR imaging.

**Figure 8:**
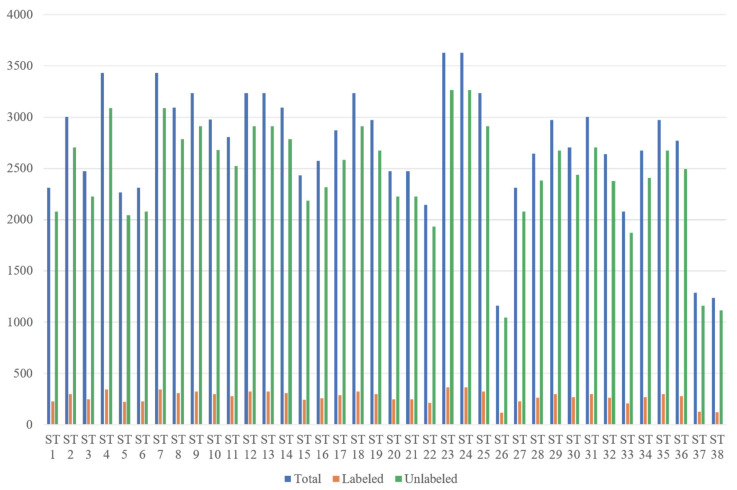
Sample distribution of the 38 classes of space targets.

The dataset comprises 103,026 ISAR images across 38 target classes. Only 10% of the data in each class is annotated, while the remaining 90% is unlabeled. The sample distribution for each class is shown in Figure 8. Of the annotated data, 20% is fixed as the test set. From the annotated images in the training set, 10%, 20%, 50%, and 80% of the samples are randomly selected to retain their pixel-level labels and serve as labeled data for supervised training; the labels of the remaining training samples are masked and used as unlabeled data for semi-supervised learning. That is, the proportions of labeled data in the training set are 1%, 2%, 5%, and 8% of the total data, respectively, while the test set accounts for 2% of the total data. The same random seed is used for training set partitioning under each labeling ratio to ensure reproducibility of the experiments.

**Figure 9:**
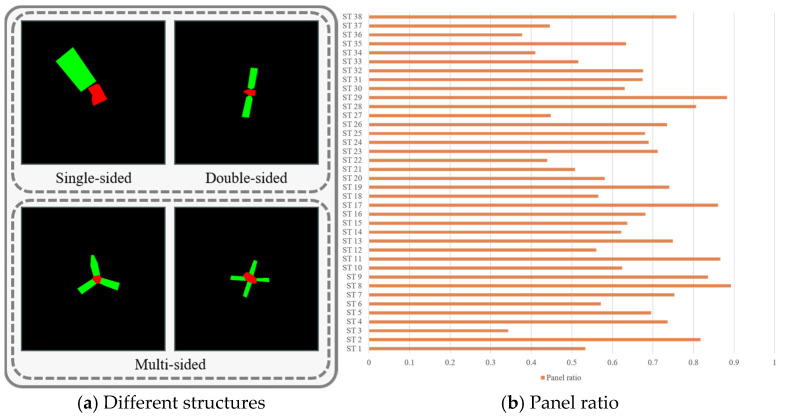
Mask-based structural illustration of different targets. (**a**) Morphological differences in solar panels across different targets. (**b**) Area proportions of solar panels across different targets.

Due to the differing structures of multiple target classes—with some payloads located outside the main body while others are inside and generally smaller in size—the components to be segmented are divided into two categories according to the common structural characteristics of the space targets in the dataset: the main body and solar panels. Target parts that are not solar panels are classified as the main body. Based on panel differences, solar panels can be further divided into single-sided solar panels and multi-sided solar panels. The variations among solar panels of different space targets are illustrated in Figure 9, which displays the range of geometric shapes and relative areas of panels present in the dataset, highlighting the diversity of target types.

It is worth noting that there are certain differences between simulated ISAR data and real ISAR data. Real ISAR data often include more complex defocusing and defects; therefore, when applying a model trained on simulated datasets to real ISAR data, appropriate processing is required.

### 4.2. Experimental Details and Evaluation Metrics

(1) Experimental environment and parameter configuration: All experiments in this paper were conducted on a unified hardware platform to ensure the comparability of results. Specifically, a server equipped with four NVIDIA GeForce RTX 4090 GPUs was used. The software environment was built on PyTorch 2.7.0 with CUDA 12.6, and Python 3.10 was used as the programming language. The random seed was set to 13 across all experiments to ensure reproducibility. Throughout the training and testing process, model weights were saved and loaded in FP32 precision. The model was trained with the AdamW optimizer with the initial learning rate set to 5 × 10^−5^ and the weight decay coefficient set to 1 × 10^−2^. The model was trained for 100 epochs with a batch size of 6.

Model parameters were set as follows: the number of encoder layers in Section 3.1 was set to 12, and the number of upsampling modules in the decoder was set to 4; the number of feature streams N in Section 3.2 was set to 4; the number of retained channels K in Section 3.3.1 was set to 100.

(2) Evaluation Metrics: To comprehensively evaluate model performance, this paper adopts Overall Accuracy (OA), mean Intersection over Union (mIoU), and F1 score as the core evaluation metrics. OA measures the global classification accuracy; IoU measures the degree of overlap between the predicted mask and the ground truth, and mIoU is the average IoU across all segmentation categories of space targets; the F1 score provides a comprehensive assessment of recall and precision for each category, and mF1 is the average F1 score across all segmentation categories of space targets. The definitions of these metrics are as follows:

(10) $$OA=\frac{TP+TN}{TP+TN+FP+FN}$$

(11) $$IoU=\frac{TP}{TP+FP+FN}$$

(12) $$mIoU=\frac{1}{N}\sum_{i=1}^{N}IoU_{i}$$

(13) $$\mathrm{Precision}=\frac{TP}{TP+FP}$$

(14) $$\mathrm{Recall}=\frac{TP}{TP+FN}$$

(15) $$F1score=2\times \frac{\mathrm{Precision}\times \mathrm{Recall}}{\mathrm{Precision}+\mathrm{Recall}}$$

(16) $$mF1==\frac{1}{N}\sum_{i=1}^{N}{F1score}_{i}$$

where N=3 in this article denotes three categories (body, panel and background), TP, TN, FP, and FN denote the true positive, true negative, false positive, and false negative.

### 4.3. Comparative Experiments

To objectively evaluate the advancement of the proposed method, this paper selects eight representative semantic segmentation methods as comparison baselines, covering mainstream architectures such as CNN, Transformer, and Mamba. The comparison methods include: UNet [53], DeepLabV3+ [54], CCASeg [55], SegFormer [56], Mask2Former [57], EoMT [58], MambaVision [59], and SegMAN [60]. These methods are based on different single architectures or combinations of multiple architectures, encompassing the mainstream technical routes in the field of semantic segmentation, thereby forming a comprehensive and challenging comparison benchmark.

UNet adopts a classic encoder–decoder architecture and effectively fuses low-level and high-level features through symmetric downsampling and upsampling paths as well as skip connections. DeepLabV3+ integrates Atrous Spatial Pyramid Pooling (ASPP) with an encoder–decoder structure, and leverages depthwise separable convolutions to capture multi-scale context while accurately recovering object boundaries. CCASeg designs a decoder based on Convolutional Cross-Attention (CCA) and employs convolutional kernels of different sizes to efficiently model local and global contexts, and enhances the recognition capability for objects of different scales by hierarchically combining multi-level features. SegFormer proposes a position-encoding-free hierarchical Transformer encoder and a lightweight all-MLP decoder, which simultaneously leverage the advantages of local and global attention by aggregating multi-layer features, and achieves simple yet efficient semantic segmentation. Mask2Former introduces a masked attention mechanism that restricts cross-attention to the foreground regions of predicted masks within the Transformer decoder, and combines multi-scale high-resolution features. EoMT re-explores the potential of ViT and discards task-specific components such as mask decoders, and proposes a mask annealing strategy to maintain segmentation accuracy while improving inference speed. MambaVision is a hybrid Mamba-Transformer visual backbone that enhances global context modeling capability through redesigned Mamba blocks and a self-attention layer in the final stage. SegMAN proposes the LASS module that integrates local attention with the State Space Model (SS2D) and a Mamba-based decoder for multi-scale context extraction, and achieves efficient global, local, and multi-scale feature modeling.

All comparison methods are retrained under the same experimental environment using the hyperparameters provided in Section 4.2 to ensure the comparability and fairness of the experimental results. In this part, we analyze the performance of the models on the dataset in detail. Through systematic quantitative comparison, we evaluate the performance of the models on various key metrics in the form of “mean ± standard deviation” across the 38 target classes. Furthermore, we also conduct qualitative analysis to further validate the effectiveness of the proposed method in the ISAR image segmentation task. These comprehensive evaluation results demonstrate the effectiveness of the proposed method under limited labeling conditions.

Table 1, Table 2, Table 3 and Table 4 present the quantitative comparison results of all methods under annotation ratios of 1%, 2%, 5%, and 8%, respectively. It can be observed that SSPNet achieves the best performance across all key metrics under different annotation ratios. A comparison of the metric changes in each method as the annotation ratio varies shows that the advantage of SSPNet becomes more pronounced as the amount of labeled data decreases. If we take the most challenging annotation ratio of 1% as an example, SSPNet achieves an mIoU of 90.59%, which is 2.07% higher than that of the second-ranked Mask2Former (88.52%). When the annotation ratio increases to 8%, the mIoU of SSPNet further improves to 91.92%, while the second-best method, CCASeg, reaches 91.78%; although the gap narrows, SSPNet still maintains its lead. Notably, the performance of SSPNet at the 1% annotation ratio is already comparable to or even better than that of most comparison methods at the 5% or even 8% annotation ratio.

An analysis of the per-class IoU and F1 scores shows that all methods achieve excellent performance on the background category, which is a natural consequence of the high proportion of background in this task. Therefore, discriminative performance is mainly reflected in the two foreground categories, i.e., body and panel. SSPNet demonstrates leading advantages in both categories. At the 1% annotation ratio, SSPNet achieves a body IoU of 85.26%, which is 3.32% higher than the second-best Mask2Former (81.94%); its panel IoU reaches 86.82%, an improvement of 2.83% over Mask2Former (83.99%). This reflects that the component geometry and edge knowledge injected by the PGM effectively constrain the segmentation shapes and alleviate mis-segmentation and boundary blurring caused by target attitude variations.

Across all annotation ratios, SSPNet consistently exhibits the lowest or near-lowest standard deviations on key metrics among the compared methods. For instance, the standard deviations of mIoU at annotation ratios of 1%, 2%, 5%, and 8% are 2.95%, 2.76%, 2.44%, and 2.45%, respectively. In contrast, some methods such as SegMAN, EoMT, and SegFormer show relatively large standard deviations at low annotation ratios. The low standard deviation exhibited by SSPNet fully demonstrates that the GMHC-ViT encoder, by broadening the feature space through manifold-constrained hyper-connections, provides more comprehensive coverage of scattering variations across different target classes.

The above results show that the CNN-based UNet and DeepLabV3+ perform moderately and lack the capability to capture global scattering relationships and structural priors. Among the Transformer-based methods represented by SegFormer, Mask2Former, and EoMT, Mask2Former performs well due to its masked attention and multi-scale high-resolution feature design, but its unified architecture does not explicitly exploit structural knowledge of space targets. Although EoMT simplifies the decoder design, it discards local detail enhancement and multi-scale interaction, and results in insufficient discriminative power for tasks demanding high boundary accuracy in ISAR images. As for the Mamba-related methods, MambaVision and SegMAN do not exhibit particular advantages on ISAR data, and their performance deteriorates severely, especially at low annotation ratios, which may be attributed to the incompatibility of their state-space modeling approach with the scattering spatial distribution of ISAR images.

In summary, the quantitative experiments fully demonstrate that SSPNet achieves efficient utilization of limited labeled data and, through structural prior guidance, significantly improves the accuracy, robustness, and multi-category generalization capability of component segmentation, and exhibits more prominent advantages especially under extremely low annotation conditions.

**Figure 10:**
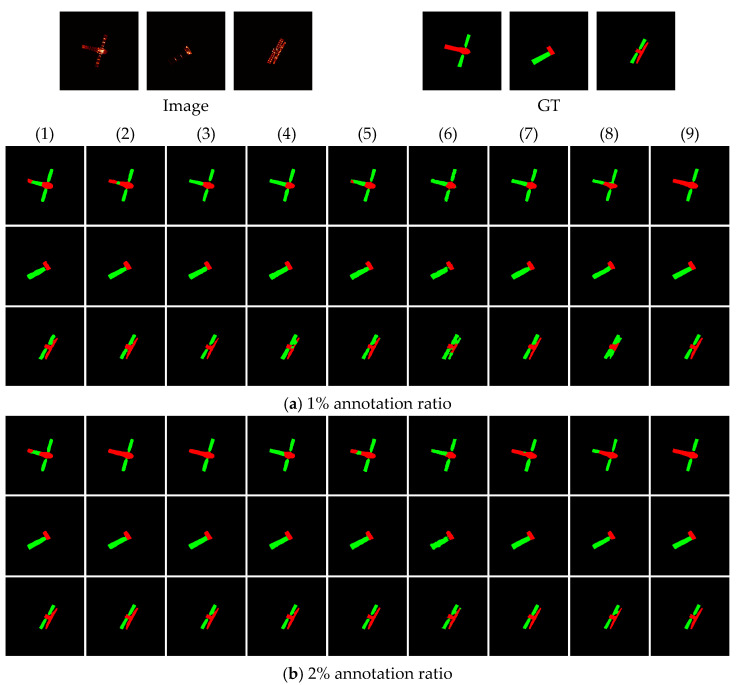

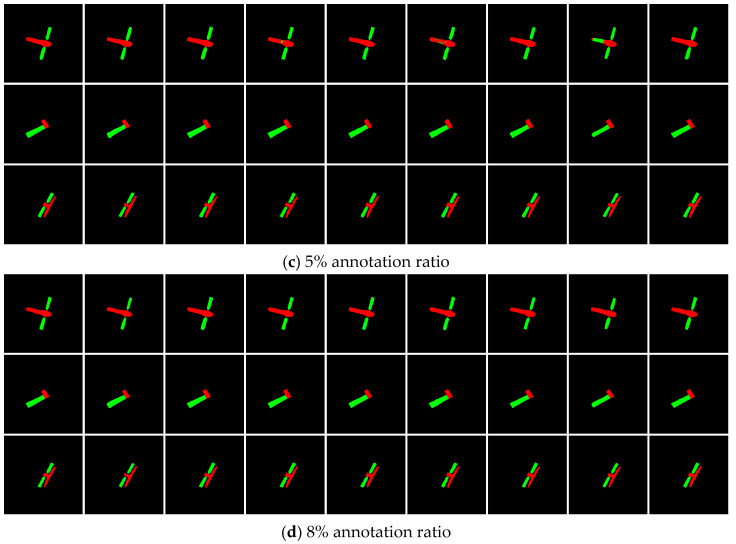
Comparison of segmentation results on representative samples. (1) through (9) represent UNet, DeepLabV3+, CCASeg, SegFormer, Mask2Former, EoMT, MambaVision, SegMAN, and the proposed SSPNet, respectively.

To intuitively evaluate the performance advantages of SSPNet, Figure 10. Comparison of segmentation results on representative samples. (1) through (9) represent UNet, DeepLabV3+, CCASeg, SegFormer, Mask2Former, EoMT, MambaVision, SegMAN, and the proposed SSPNet, respectively. provides a visual comparison of segmentation results from different methods on representative samples. It can be seen that as the amount of labeled data decreases, misclassification and edge blurring in comparison methods significantly intensify, while SSPNet consistently maintains good performance.

Although the main innovation of this paper does not target the semi-supervised learning paradigm itself, the specifically designed perturbation strategy motivates a comparison between the proposed method and the two most relevant semi-supervised methods—FixMatch and Unimatch. To ensure fairness of the comparison, the backbones used by FixMatch and Unimatch are kept consistent with those of SSPNet, and only the perturbation strategy is replaced. Due to space limitations, we select only a moderate annotation ratio (i.e., 5%) for the experiments.

**Figure 11:**
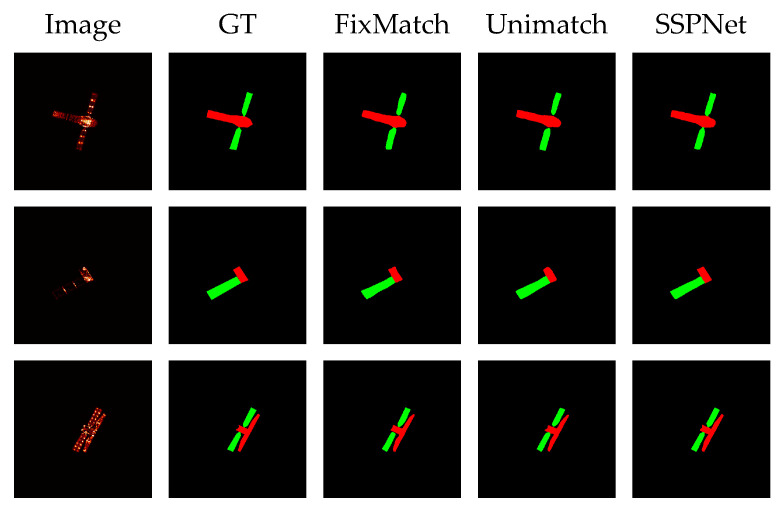
Comparison results with semi-supervised learning methods.

The quantitative comparison results are presented in Table 5. The experimental results show that SSPNet achieves an mIoU of 91.85%, which is 1.10% higher than FixMatch and 1.11% higher than Unimatch, and maintains consistent leadership in other metrics. In particular, for the two foreground categories of body and panel, SSPNet exhibits lower IoU standard deviations than the two compared methods, thereby demonstrating stronger category stability. The reason for this advantage is that SSPNet, through the tailored perturbation strategy, is able to construct meaningful multi-views without disrupting the relative intensity relationships of scattering centers, thereby providing more reliable supervisory signals for consistency constraints; in contrast, FixMatch and Unimatch generally adopt optical perturbations, which easily destroy the scattering semantics in ISAR images, thereby causing the consistency loss to misguide the model. The visualization results are shown in Figure 11. Comparison results with semi-supervised learning methods.

### 4.4. Efficiency Comparison

In addition to model accuracy, model efficiency is also a key dimension for evaluating model performance. Parameter count (Params), floating point operations (FLOPs) and inference time are the core metrics in this regard. Specifically, Params reflect the spatial complexity and training requirements of the model, while FLOPs reflect the temporal complexity and computational cost; together, they form a quantitative evaluation system for model efficiency. To ensure experimental consistency, we conducted the model efficiency evaluation under the same hardware environment as the accuracy experiments in Section 4.3. Since Params and FLOPs do not vary with the annotation ratio, we only present the model accuracy at a 5% annotation ratio to more clearly compare the accuracy changes corresponding to different Params and FLOPs. Inference time refers to the average inference time per sample, i.e., the mean of the inference times over all samples in the test set.

**Figure 12:**
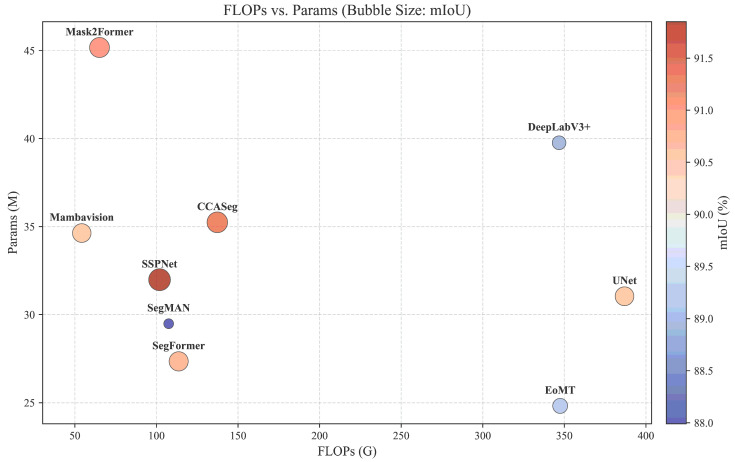
FLOPs–Params scatter plot. The horizontal axis represents FLOPs (G), and the vertical axis represents Params (M). The bubble size is proportional to the mIoU (%).

Table 6 systematically presents the quantitative comparison results of model efficiency for different methods. Figure 12 intuitively illustrates the trade-off between accuracy and efficiency for different methods through a FLOPs–Params scatter plot. Since the trend of the mF1 metric across different methods is largely consistent with that of mIoU, we adopt only the mIoU metric to simplify the illustration.

In terms of parameter count, SSPNet has 31.98M parameters, which places it at a moderately light level among all compared methods. Specifically, its parameter count is significantly lower than that of Mask2Former (45.17M), roughly comparable to that of UNet (31.04M), and slightly higher than that of SegFormer (27.35M) and EoMT (24.82M). Although the SSPNet encoder introduces additional multi-feature streams and gating mechanisms, and the PGM includes parallel shape and edge feature extraction branches, the parameter count does not inflate despite the improvement in model accuracy. The main reasons are that GMHC-ViT shares some of the base parameters through its manifold-constrained design, and that the edge and shape branches in the PGM both adopt lightweight depthwise separable convolutions and a smaller ViT module, thereby effectively reducing parameter redundancy.

In terms of computational cost, SSPNet requires 101.90G FLOPs, which is far lower than the FLOPs of UNet (386.88G), DeepLabV3+ (346.78G), and EoMT (347.44G). In comparison with SegMAN (107.50G) and SegFormer (113.62G), SSPNet is also competitive in FLOPs, thereby indicating its advantage of low computational burden during inference. It should be noted that although Mask2Former (65.10G) and MambaVision (54.22G) have lower FLOPs, the accuracy data in Table 6 show that SSPNet leads these two models by 0.8% and 1.32% in mIoU, respectively. Figure 12 intuitively illustrates this accuracy-efficiency trade-off: SSPNet occupies the position with the largest bubble in the lower-left (low FLOPs, low Params) region, indicating that it achieves higher segmentation performance at a lower computational cost, thereby striking a better balance between accuracy and efficiency.

The reasons for SSPNet’s efficiency advantage are mainly the following three points. First, although the GMHC-ViT encoder broadens the feature space through parallel feature streams, its inter-stream mixing and gating operations incur only limited computational overhead, and its powerful feature representation capability reduces the reliance on a deep decoder. Second, the CSM achieves channel selection with lightweight global average pooling and a single-layer MLP, thereby incurring almost no additional computational burden. Third, the PGM only reshapes features of weakly activated channels without increasing the spatial resolution of feature maps, and the decoder is entirely composed of several simple upsampling layers, thereby avoiding skip connections, multi-scale fusion, and other operations that typically consume substantial computational resources. Therefore, although SSPNet integrates multiple modules, its overall computational efficiency remains at a level comparable to or even better than current mainstream models. However, in terms of inference time, the performance of SSPNet is not ideal. A possible reason is that its structural priors are loaded independently of the ISAR image input, and the computational efficiency difference between the prior feature extraction branch and the encoding feature extraction branch leads to a significant increase in inference time.

### 4.5. Ablation Experiments

To systematically verify the contributions and synergistic effects of each core module of SSPNet on overall performance, we design a series of ablation experiments in this section. The main advantages of SSPNet lie in the GMHC-ViT encoder and the PGM. The GMHC-ViT broadens the feature space to enhance cross-category generalization, while the PGM extracts structural prior features to enhance the model’s perception of structural semantics. Therefore, ablation experiments in this section focus on these two components. In addition, we also design specific semi-supervised perturbation strategies; hence, we also conduct brief ablation experiments on these strategies to verify their effectiveness. All ablation experiments are performed under the same hardware environment and hyperparameter settings, with only the ablated component varied, to ensure fairness. All experiments in this section are conducted at a 5% annotation ratio. Since the trends of OA, mIoU, and mF1 metrics are largely consistent in this section, we adopt only the most representative mIoU as the primary quantitative evaluation metric.

#### 4.5.1. Experimental Analysis of GMHC-ViT

**Figure 13:**
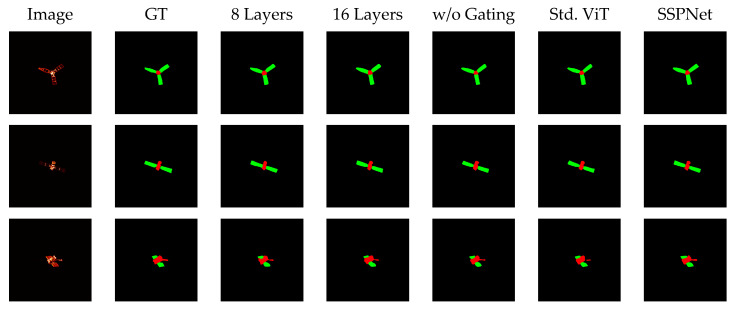
Segmentation results of different encoder configurations on representative samples. ‘Std. ViT’ = ‘Standard ViT’.

To validate the effectiveness of the key design elements in the GMHC-ViT encoder, this section conducts ablation experiments from three aspects: the encoder depth configuration, the necessity of the gating mechanism, and the architectural comparison with the standard ViT. All ablation variants keep other components unchanged, with only the ablated part varied. The complete model in this paper, whose encoder has 12 layers, serves as the comparison baseline. Table 7 presents the quantitative results under different parameter configurations. Figure 13 shows the visualization results.

**Figure 14:**
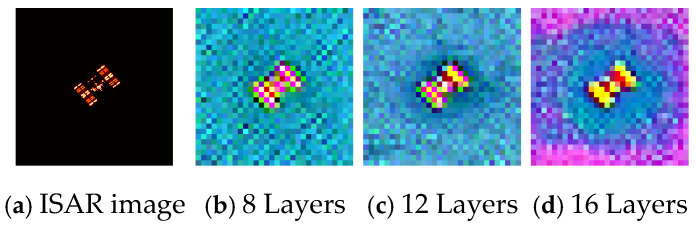
PCA dimensionality reduction results of encoder features with different numbers of layers. All channels are used for dimensionality reduction and visualization.

**Figure 15:**
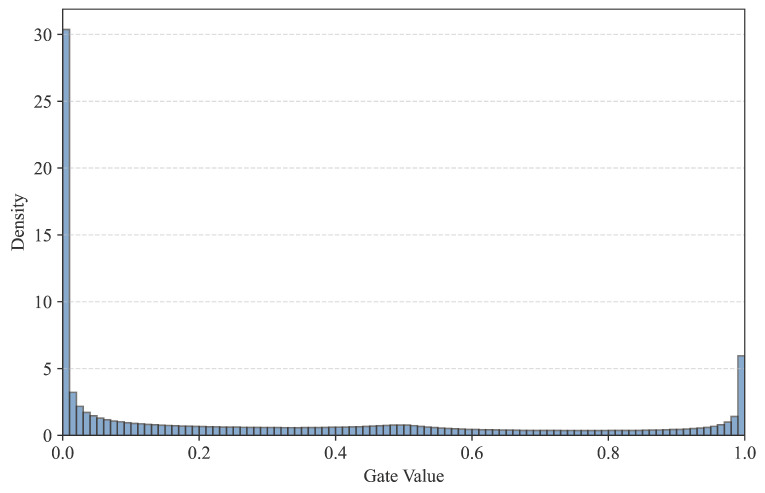
Histogram of gating weight distribution.

**Figure 16:**
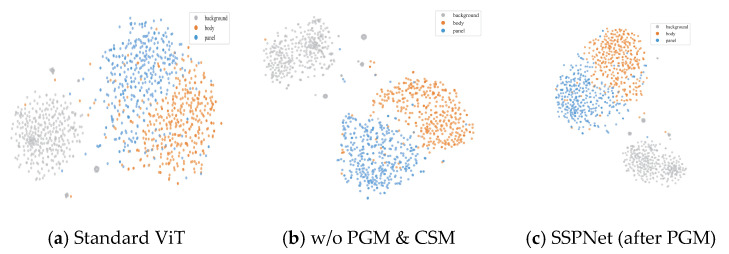
t-SNE dimensionality reduction results. (**a**) shows the dimensionality reduction result of the standard ViT encoder features; (**b**) shows the dimensionality reduction result of the GMHC-ViT encoder features; (**c**) shows the dimensionality reduction result after prior information injection into the GMHC-ViT encoder features.

(1) Impact of the number of encoder layers. After the number of GMHC-ViT encoder layers was reduced to 8 or expanded to 16, the mIoU metric declined in both cases: the mIoU was 91.48% with 8 layers, and dropped to 91.36% with 16 layers. The possible reasons are as follows. The performance drop with 8 layers can be attributed to insufficient model capacity: a shallow encoder struggles to adequately model the diversity in scattering distribution and geometric morphology across the 38 classes of space targets. With 16 layers, although the number of parameters increased to 40.57M (a 26.86% increase), the mIoU of the 16-layer model was actually slightly lower than that with 8 layers, thereby indicating that an overly deep network may be more prone to overfitting under limited labeled data, even with the additional regularization provided by a large amount of unlabeled data, and that overly deep feature transformations may weaken the effectiveness of structural prior injection. Figure 14 further presents the visualization results of feature maps at the output of the three encoder depths for the same input image: the 8-layer features exhibit significant noise in component boundary regions, and transitions between the solar panel and the main body are blurred; the 16-layer features show substantial background noise and over-focusing of feature responses in certain local regions; the 12-layer features display clear component contours and consistent foreground activation, thereby corroborating the quantitative findings.

(2) Role of the gating mechanism. After the gating mechanism was removed from GMHC-ViT, the model’s mIoU dropped by 0.38%. Notably, the standard deviation increased from ±2.44% to ±2.85%, thereby indicating that the gating-free multi-stream fusion exhibits unstable performance on some extreme samples. Figure 15 illustrates the distribution of gating weights: the Sigmoid activation values output by the gating are distributed continuously between 0 and 1, with a mean of 0.32, while certain channels exhibit high activation (>0.95) or low activation (<0.05). This distribution implies that the gating is not a simple identity mapping; rather, it dynamically assigns differentiated weights to different channels based on the input in each forward pass. This adaptive selection mechanism enables information refinement in multi-stream features while preserving diversity, thereby achieving a stable improvement and better inter-class consistency at a very small parameter cost (3.54M).

(3) Comparison with the standard ViT. Replacing the GMHC-ViT with a ViT of the same depth led to a 1.61% decrease in mIoU, which was also lower than that of the GMHC-ViT without the gating mechanism. This indicates that the single-stream representation of the standard ViT is limited in its capacity to capture the diverse scattering patterns of multi-class targets, whereas the GMHC-ViT broadens the representation space through parallel multi-feature streams, and even without the gating mechanism, richer features can be obtained via inter-stream mixing. In other words, the multi-stream structure establishes the foundation for feature diversity, upon which the gating mechanism accomplishes adaptive feature refinement. Panels (a) and (b) in Figure 16 present the t-SNE dimensionality reduction visualization results of features from different encoders: for the standard ViT, the feature points of the three classes can be roughly distinguished, but there is no clear separation boundary between body and panel, and the feature points within each class are relatively scattered; in the visualization of the GMHC-ViT, by contrast, the feature points of the three classes each form a distinct cluster, with obvious inter-class gaps; especially, the confusion region between body and panel is significantly reduced. This further demonstrates the effectiveness of the multi-stream architecture.

#### 4.5.2. Experimental Analysis of PGM

To validate the roles of the PGM and its associated channel screening mechanism CSM, we design two groups of ablation experiments in this section: one involving entirely removing the PGM and CSM, and the other involving adjusting the number of retained channels K in the CSM. Table 8 presents the quantitative results under each configuration.

(1) Analysis of the role of PGM: When the CSM and PGM are completely removed, the encoder output features are directly fed into the decoder. The mIoU drops by 1.32%, while the standard deviation increases from ±2.44% to ±3.31%, thereby indicating a decline in performance stability. This shows that even though the GMHC-ViT possesses strong feature extraction capability, a considerable proportion of weakly activated channels still exist in its encoded features, and these channels lack sufficient discriminative semantics. The PGM reshapes the encoded features by injecting structural priors, thereby significantly improving segmentation accuracy. Panels (b) and (c) in Figure 16 show the dimensionality reduction visualization results of the encoded features before and after structural prior injection. Without the PGM, although the feature points of different classes form their own clusters with clear separation boundaries, the feature points within each cluster are relatively scattered; after prior injection, the distribution within each feature cluster becomes tighter, while distinct inter-class boundaries still exist, thereby indicating that the structural prior information enhances the model’s discriminability for different components. This visualization result intuitively demonstrates the effectiveness of prior injection.

(2) Influence of K in CSM: When K is 50, mIoU decreases by 0.57%; when K is 150, mIoU decreases by 0.39%. The possible reasons for these results are as follows: when K is too small, only a very few high-response channels are retained, and a large number of channels need to be reconstructed via priors. Over-reliance on structural priors constrains the feature representation near typical component geometry templates, thereby weakening the adaptability to variations in scattering models caused by attitude changes in actual ISAR images, and thus leading to a decline in accuracy. When K is too large, the vast majority of channels are judged as strong responses and directly retained, with only a small number of channels receiving prior enhancement. In this case, the scope of the PGM is narrowed, and the guiding power of structural priors is diluted, thereby resulting in performance degradation. Therefore, an appropriate K value is required to balance the relative relationship between strong and weak channels, so as to achieve optimal performance.

The above results demonstrate the effectiveness of the co-design of PGM and CSM. The CSM adaptively locates the weakly semantic channels that require enhancement, thereby avoiding the coverage of all channels or information dilution by prior knowledge; the PGM establishes fine-grained semantic associations between weak channel features and structural priors via cross-attention, thereby achieving targeted feature reshaping. This “evaluation–filtering–enhancement” chain enables the effective integration of independent priors into the segmentation process, thereby improving boundary accuracy and structural integrity in multi-category space target segmentation.

#### 4.5.3. Experimental Analysis of Perturbation Strategy

**Figure 17:**
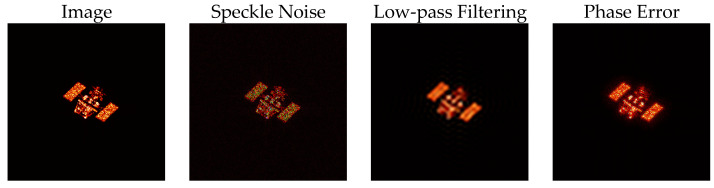
Effect of different perturbation strategies.

To evaluate the contribution of the strong perturbation strategy tailored to ISAR image characteristics for semi-supervised learning, this section compares multiple perturbation configurations. The configuration adopting all three perturbation strategies serves as the baseline, and each variant removes only one of the three perturbation strategies while keeping the other two unchanged. Table 9 presents the quantitative results under each configuration. Figure 17 illustrates the effects of different perturbation strategies.

Removing low-pass filtering resulted in the largest performance drop, with mIoU declining from 91.85% to 91.48%, corresponding to a decrease of 0.37%. Low-pass filtering blurs ISAR images by truncating high-frequency components in the frequency domain, thereby forcing the model to strengthen its ability to recover high-frequency boundary details during semi-supervised learning. Without this perturbation operator, the model’s discriminative power for component edges is weakened, thus affecting segmentation accuracy.

Removing multiplicative speckle noise caused an mIoU drop of 0.14%. This perturbation operator simulates intensity fluctuations induced by coherent speckle, thereby enabling the model to learn robustness to intensity variations during consistency regularization. After its removal, the model’s segmentation stability in low-contrast regions declines, thereby leading to local mis-segmentation and reduced stability.

Removing frequency-domain phase error caused an mIoU drop of 0.06%. This perturbation applies quadratic phase modulation, resulting in structural blurring and detail degradation. Its impact is relatively small, possibly because the global contours of components can still be effectively constrained by the structural priors, and the degree of degradation is comparatively mild.

Overall, the random combination of the three perturbation operators enables the model to maintain effective perception of various degradation modes, thereby achieving optimal performance and stability. This demonstrates the importance of diverse, targeted strong perturbation strategies for semi-supervised learning.

### 4.6. Robustness to Noise

**Figure 18:**
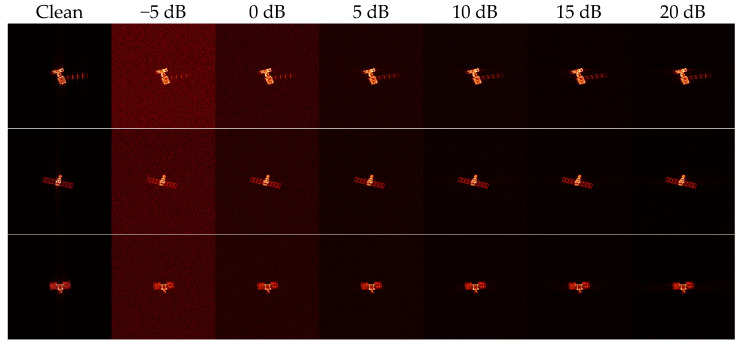
Examples of ISAR images under different SNRs (–5 dB to 20 dB).

In real-world scenarios, radar echoes are inevitably affected by noise, which causes intensity fluctuations in scattering centers and blurred component edges, thereby degrading segmentation accuracy. However, most existing methods are validated only under clean or high-SNR conditions and lack evaluation of model performance under low-SNR conditions. To systematically assess the robustness of SSPNet under varying SNR conditions, this section adds Gaussian white noise of different intensities to the ISAR echoes of three typical target classes in the dataset, with SNR settings ranging from −5 dB to 20 dB, which comprise six levels in total. The noisy echoes are then processed to produce noisy ISAR images, as shown in Figure 18. All experiments in this section are conducted at a 5% annotation ratio, and the relevant metrics are computed only on these three typical target classes.

Table 10 presents the experimental results under different SNR conditions. SSPNet consistently maintains stable high-precision segmentation across the SNR range from −5 dB to the clean signal. The background class IoU exceeds 99.84% under all noise conditions, almost unaffected by noise; this stems from the naturally strong contrast between target and background in ISAR images. For the two foreground classes, body IoU gradually decreases from 91.46% (clean) to 85.92% at −5 dB, and panel IoU decreases from 92.90% to 89.75%; the corresponding mIoU drops only from 94.75% to 91.84%, remaining above 91.8% even at the extremely low SNR of −5 dB.

From the overall trend, as the SNR increases, all core metrics essentially exhibit a monotonic upward trend, but exhibit a characteristic of diminishing marginal gains: mIoU increases by 0.30% from −5 dB to 0 dB and by only 0.09% from 0 dB to 10 dB, while performance nearly saturates above 10 dB. This suggests that under higher SNR conditions, the noise level is not the primary bottleneck limiting segmentation accuracy. The most notable change occurs when noise is completely removed: mIoU jumps from 92.32% at 20 dB to 94.75%, an increase of 2.43%. This phenomenon indicates that even slight noise can submerge the weak scattering details inside components and disrupt the subtle intensity transitions near boundaries, whereas under noise-free conditions, the intact scattering distribution enables the model to fully exploit all discriminative information, thereby achieving higher segmentation accuracy.

The strong noise robustness of SSPNet can be attributed to two aspects: first, the structural priors injected by the PGM are independent of ISAR image quality, so even when images degrade, the priors can still provide geometric and boundary constraints; second, the perturbation strategy adopted during semi-supervised training exposes the model to diverse degradation patterns at the training stage, thereby endowing it with stronger anti-interference capability.

**Figure 19:**
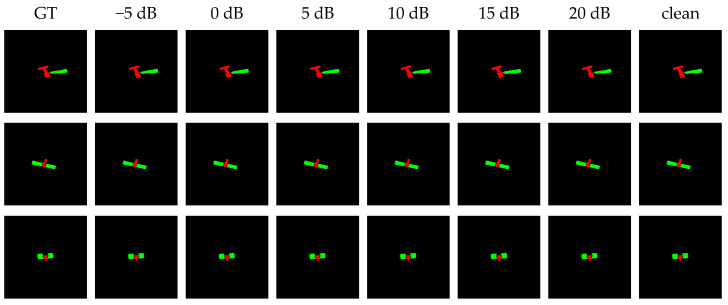
Comparison of segmentation results on representative samples under different SNRs.

Figure 19 intuitively illustrates the segmentation results of samples under different SNRs. As the SNR decreases, edge blurring in segmentation gradually increases, with evident misclassification occurring especially at the turning points of component boundaries. This indicates that the introduction of noise causes certain interference to the localization of weak scattering points along the boundaries.

## 5. Conclusions

This paper addresses the three major challenges facing component-level semantic segmentation of space target ISAR images—the scarcity of annotated data, the absence of structural priors for components, and the morphological diversity of multi-category targets—by proposing SSPNet, a semi-supervised structural prior-guided network. In terms of model architecture, the GMHC-ViT encoder is designed to broaden the feature representation space through parallel multi-feature streams and a gating mechanism, thereby effectively enhancing the generalization capability for the complex scattering patterns of multi-class space targets. Additionally, the PGM is innovatively proposed, which leverages shape and edge priors extracted from two-dimensional projections of CAD models to adaptively enhance the weakly activated channels of the encoded features and explicitly inject geometric structural knowledge independent of image quality into the segmentation process. In terms of training strategy, a strong perturbation strategy tailored to ISAR images is designed, thereby enabling the semi-supervised consistency regularization framework to effectively mine component semantic information from large amounts of unlabeled data.

Comprehensive evaluations on an ISAR image dataset of 38 classes of space targets demonstrate that SSPNet outperforms mainstream segmentation methods such as UNet, DeepLabV3+, SegFormer, and Mask2Former, as well as classic semi-supervised methods such as FixMatch and UniMatch, under different annotation ratios (1% to 8%). Moreover, SSPNet exhibits strong robustness under varying SNR conditions, and the ablation experiments also sufficiently validate the effectiveness of the core designs, including the multi-stream gated architecture, the prior injection mechanism, and the perturbation strategy adapted to ISAR characteristics.

The selection of the K value in CSM is not fixed; it should be chosen according to the characteristics of the dataset and the task. The parameter ranges adopted by the different perturbation operators in Section 3.4 are selected based on their functional characteristics and manual experience. The adaptive selection of these hyperparameters is a direction worth investigating. Although SSPNet achieves excellent performance on simulated datasets, it still has some limitations. For example, the structural priors come from simplified CAD models, which may not match space targets with unusual configurations, thus failing to effectively guide the segmentation process. The segmentation performance also degrades under severe occlusion and extreme pose scenarios. Moreover, the model has not been validated on real data. In future work, we plan to verify the method on measured ISAR data, investigate an adaptive prior update mechanism for unknown targets, and extend it to cover more component categories.

## Figures and Tables

**Table 1:** Experimental results with an annotation ratio of 1% (Unit: %). bkg. = background.

Method	IoU per Category			F1score per Category			Overall Metrics		
	Bkg.	Body	Panel	Bkg.	Body	Panel	OA	mIoU	mF1
Sup. only	99.60 ± 0.14	78.80 ± 8.37	80.73 ± 9.05	99.80 ± 0.07	87.55 ± 5.93	88.66 ± 6.64	99.49 ± 0.24	86.38 ± 5.44	92.00 ± 3.94
UNet	99.61 ± 0.14	80.31 ± 7.22	81.72 ± 7.26	99.80 ± 0.07	88.60 ± 5.01	89.45 ± 5.17	99.52 ± 0.19	87.21 ± 4.39	92.62 ± 3.10
DeepLabV3+	99.61 ± 0.13	80.77 ± 7.22	81.54 ± 7.03	99.80 ± 0.07	88.90 ± 4.98	89.35 ± 4.99	99.53 ± 0.19	87.31 ± 4.29	92.68 ± 3.02
CCASeg	99.63 ± 0.12	80.60 ± 7.46	82.78 ± 6.45	99.81 ± 0.06	88.74 ± 5.16	90.18 ± 4.45	99.56 ± 0.16	87.67 ± 4.18	92.91 ± 2.90
SegFormer	99.62 ± 0.14	73.23 ± 12.75	78.57 ± 11.56	99.81 ± 0.07	83.11 ± 10.31	87.15 ± 8.48	99.42 ± 0.34	83.81 ± 7.79	90.02 ± 5.97
Mask2Former	99.63 ± 0.12	81.94 ± 6.92	83.99 ± 6.20	99.82 ± 0.06	89.66 ± 4.71	90.92 ± 4.34	99.58 ± 0.16	88.52 ± 3.94	93.46 ± 2.74
EoMT	99.58 ± 0.15	73.08 ± 12.14	77.29 ± 11.75	99.79 ± 0.08	83.44 ± 9.29	86.34 ± 8.89	99.40 ± 0.34	83.32 ± 7.55	89.86 ± 5.72
MambaVision	99.63 ± 0.13	78.20 ± 10.33	80.88 ± 10.21	99.81 ± 0.07	87.01 ± 7.41	88.76 ± 7.41	99.49 ± 0.30	86.23 ± 6.56	91.86 ± 4.76
SegMAN	99.48 ± 0.17	67.85 ± 16.02	72.52 ± 11.75	99.74 ± 0.08	78.78 ± 14.24	83.12 ± 9.12	99.31 ± 0.31	79.95 ± 8.69	87.21 ± 7.17
**SSPNet**	**99.68 ± 0.12**	**85.26 ± 6.04**	**86.82 ± 4.04**	**99.84 ± 0.06**	**91.75 ± 4.10**	**92.73 ± 2.60**	**99.65 ± 0.12**	**90.59 ± 2.95**	**94.77 ± 1.97**

**Table 2:** Experimental results with an annotation ratio of 2% (Unit: %). bkg. = background.

Method	IoU per Category			F1score per Category			Overall Metrics		
	Bkg.	Body	Panel	Bkg.	Body	Panel	OA	mIoU	mF1
Sup. only	99.64 ± 0.13	82.56 ± 6.37	84.35 ± 6.06	99.82 ± 0.06	90.06 ± 4.34	91.16 ± 4.13	99.58 ± 0.15	88.85 ± 3.68	93.68 ± 2.53
UNet	99.63 ± 0.12	82.87 ± 6.39	84.40 ± 5.26	99.82 ± 0.06	90.28 ± 4.35	91.26 ± 3.49	99.59 ± 0.14	88.97 ± 3.40	93.78 ± 2.31
DeepLabV3+	99.65 ± 0.12	84.17 ± 5.87	85.33 ± 4.48	99.82 ± 0.06	91.11 ± 3.92	91.83 ± 2.95	99.61 ± 0.13	89.72 ± 2.94	94.26 ± 1.96
CCASeg	99.68 ± 0.11	85.08 ± 5.74	86.42 ± 4.30	99.84 ± 0.06	91.65 ± 3.81	92.50 ± 2.70	99.64 ± 0.12	90.39 ± 2.93	94.66 ± 1.91
SegFormer	99.66 ± 0.12	81.91 ± 8.16	83.83 ± 8.14	99.83 ± 0.06	89.51 ± 5.71	90.73 ± 5.75	99.57 ± 0.22	88.47 ± 5.15	93.36 ± 3.64
Mask2Former	99.66 ± 0.11	84.05 ± 5.96	85.93 ± 4.56	99.83 ± 0.06	91.04 ± 3.95	92.18 ± 2.97	99.62 ± 0.12	89.88 ± 3.03	94.35 ± 2.01
EoMT	99.60 ± 0.15	78.76 ± 8.43	80.83 ± 8.40	99.80 ± 0.07	87.54 ± 5.90	88.88 ± 5.90	99.50 ± 0.22	86.40 ± 5.13	92.07 ± 3.58
MambaVision	99.63 ± 0.12	83.04 ± 6.52	84.13 ± 5.43	99.82 ± 0.06	90.32 ± 4.46	91.06 ± 3.65	99.59 ± 0.14	88.94 ± 3.57	93.73 ± 2.44
SegMAN	99.57 ± 0.13	78.98 ± 8.43	79.87 ± 7.85	99.79 ± 0.07	87.61 ± 6.02	88.31 ± 5.55	99.49 ± 0.19	86.14 ± 5.07	91.90 ± 3.61
**SSPNet**	**99.69 ± 0.11**	**86.40 ± 5.35**	**87.46 ± 3.90**	**99.84 ± 0.06**	**92.38 ± 3.60**	**93.11 ± 2.48**	**99.66 ± 0.12**	**91.18 ± 2.76**	**95.11 ± 1.81**

**Table 3:** Experimental results with an annotation ratio of 5% (Unit: %). bkg. = background.

Method	IoU per Category			F1score per Category			Overall Metrics		
	Bkg.	Body	Panel	Bkg.	Body	Panel	OA	mIoU	mF1
Sup. only	99.67 ± 0.12	85.67 ± 5.42	86.96 ± 3.83	99.84 ± 0.06	92.03 ± 3.51	92.84 ± 2.37	99.65 ± 0.12	90.77 ± 2.68	94.90 ± 1.71
UNet	99.67 ± 0.12	85.10 ± 5.61	87.03 ± 4.06	99.84 ± 0.06	91.68 ± 3.68	92.88 ± 2.54	99.65 ± 0.12	90.60 ± 2.75	94.80 ± 1.79
DeepLabV3+	99.63 ± 0.11	82.42 ± 7.21	84.72 ± 3.93	99.82 ± 0.06	90.02 ± 4.80	91.55 ± 2.50	99.60 ± 0.11	88.92 ± 3.25	93.80 ± 2.14
CCASeg	99.69 ± 0.11	86.69 ± 5.26	87.61 ± 3.63	99.85 ± 0.06	92.64 ± 3.41	93.23 ± 2.22	99.67 ± 0.11	91.33 ± 2.60	95.24 ± 1.66
SegFormer	99.69 ± 0.11	85.53 ± 5.43	87.02 ± 4.35	99.84 ± 0.06	91.92 ± 3.61	92.86 ± 2.77	99.65 ± 0.12	90.75 ± 2.88	94.87 ± 1.89
Mask2Former	99.68 ± 0.11	86.05 ± 5.21	87.42 ± 3.57	99.84 ± 0.06	92.28 ± 3.35	93.13 ± 2.17	99.66 ± 0.11	91.05 ± 2.51	95.08 ± 1.59
EoMT	99.65 ± 0.13	83.40 ± 6.22	84.75 ± 5.61	99.82 ± 0.07	90.62 ± 4.13	91.47 ± 3.63	99.60 ± 0.15	89.27 ± 3.48	93.97 ± 2.28
MambaVision	99.67 ± 0.11	85.27 ± 6.40	86.66 ± 3.70	99.83 ± 0.05	91.77 ± 4.23	92.67 ± 2.33	99.64 ± 0.11	90.53 ± 2.83	94.76 ± 1.86
SegMAN	99.57 ± 0.14	83.00 ± 6.84	81.40 ± 5.52	99.78 ± 0.07	90.36 ± 4.61	89.45 ± 3.84	99.54 ± 0.15	87.99 ± 3.77	93.20 ± 2.58
**SSPNet**	**99.70 ± 0.11**	**87.51 ± 4.95**	**88.33 ± 3.43**	**99.85 ± 0.06**	**93.11 ± 3.21**	**93.65 ± 2.11**	**99.68 ± 0.11**	**91.85 ± 2.44**	**95.54 ± 1.56**

**Table 4:** Experimental results with an annotation ratio of 8% (Unit: %). bkg. = background.

Method	IoU per Category			F1score per Category			Overall Metrics		
	Bkg.	Body	Panel	Bkg.	Body	Panel	OA	mIoU	mF1
Sup. only	99.69 ± 0.12	86.21 ± 5.42	87.68 ± 3.67	99.84 ± 0.06	92.35 ± 3.49	93.28 ± 2.23	99.66 ± 0.12	91.19 ± 2.65	95.16 ± 1.68
UNet	99.67 ± 0.11	84.92 ± 5.41	87.00 ± 3.57	99.83 ± 0.06	91.60 ± 3.53	92.89 ± 2.20	99.65 ± 0.12	90.53 ± 2.52	94.77 ± 1.63
DeepLabV3+	99.60 ± 0.12	79.10 ± 8.27	84.78 ± 4.10	99.80 ± 0.06	87.86 ± 5.72	91.57 ± 2.62	99.57 ± 0.12	87.82 ± 3.37	93.08 ± 2.31
CCASeg	99.71 ± 0.11	87.31 ± 5.10	88.33 ± 3.40	99.85 ± 0.05	93.00 ± 3.32	93.66 ± 2.07	99.68 ± 0.11	91.78 ± 2.49	95.50 ± 1.59
SegFormer	99.70 ± 0.11	86.80 ± 5.17	88.00 ± 3.75	99.85 ± 0.06	92.70 ± 3.36	93.45 ± 2.33	99.68 ± 0.11	91.50 ± 2.59	95.33 ± 1.67
Mask2Former	99.69 ± 0.11	86.45 ± 4.95	87.81 ± 3.28	99.84 ± 0.06	92.52 ± 3.15	93.37 ± 1.97	99.67 ± 0.11	91.31 ± 2.33	95.24 ± 1.46
EoMT	99.66 ± 0.13	84.70 ± 6.04	85.87 ± 4.80	99.83 ± 0.06	91.43 ± 3.96	92.17 ± 3.05	99.62 ± 0.14	90.08 ± 3.19	94.48 ± 2.06
MambaVision	99.68 ± 0.10	86.11 ± 5.61	86.91 ± 3.49	99.84 ± 0.05	92.30 ± 3.65	92.82 ± 2.23	99.66 ± 0.11	90.90 ± 2.54	94.99 ± 1.66
SegMAN	99.65 ± 0.11	84.96 ± 5.89	85.40 ± 3.93	99.82 ± 0.06	91.59 ± 3.94	91.93 ± 2.47	99.62 ± 0.11	90.00 ± 2.80	94.45 ± 1.84
**SSPNet**	**99.71 ± 0.11**	**87.59 ± 5.02**	**88.48 ± 3.34**	**99.85 ± 0.06**	**93.14 ± 3.28**	**93.74 ± 2.03**	**99.69 ± 0.11**	**91.92 ± 2.45**	**95.58 ± 1.57**

**Table 5:** Experimental results with an annotation ratio of 5% (Unit: %). bkg. = background.

Method	IoU per Category			F1score per Category			Overall Metrics		
	Bkg.	Body	Panel	Bkg.	Body	Panel	OA	mIoU	mF1
FixMatch	99.67 ± 0.12	85.48 ± 5.65	87.09 ± 3.61	99.84 ± 0.06	91.92 ± 3.70	92.95 ± 2.19	99.65 ± 0.12	90.75 ± 2.66	94.90 ± 1.70
Unimatch	99.67 ± 0.12	85.53 ± 5.82	87.02 ± 3.97	99.84 ± 0.06	91.94 ± 3.83	92.87 ± 2.49	99.65 ± 0.12	90.74 ± 2.86	94.88 ± 1.85
**SSPNet**	**99.70 ± 0.11**	**87.51 ± 4.95**	**88.33 ± 3.43**	**99.85 ± 0.06**	**93.11 ± 3.21**	**93.65 ± 2.11**	**99.68 ± 0.11**	**91.85 ± 2.44**	**95.54 ± 1.56**

**Table 6:** Comparison of efficiency and accuracy of different models.

Method	Params (M)	FLOPs (G)	Inference Time (ms)	mIoU (%)
UNet	31.04	386.88	13.25	90.60
DeepLabV3+	39.76	346.78	11.55	88.92
CCASeg	35.24	137.30	21.46	91.33
SegFormer	27.35	113.62	13.52	90.75
Mask2Former	45.17	65.10	19.93	91.05
EoMT	24.82	347.44	75.55	89.27
MambaVision	34.63	54.22	9.81	90.53
SegMAN	29.49	107.50	27.39	87.99
SSPNet	31.98	101.90	70.20	91.85

**Table 7:** Ablation results of the GMHC-ViT encoder. ‘w/o’ = ‘without’.

Configuration	Params (M)	FLOPs (G)	mIoU (%)
8 Layers	23.40	77.04	91.48 ± 2.68
16 Layers	40.57	126.74	91.36 ± 2.72
w/o Gating	28.44	72.84	91.47 ± 2.85
Standard ViT	27.51	71.02	90.24 ± 2.73
**SSPNet**	**31.98**	**101.90**	**91.85 ± 2.44**

**Table 8:** Ablation results of the PGM. ‘w/o’ = ‘without’.

Configuration	mIoU (%)
w/o PGM & CSM	90.53 ± 3.31
K = 50	91.28 ± 2.71
K = 150	91.46 ± 2.88
**SSPNet (K = 100)**	**91.85 ± 2.44**

**Table 9:** Experimental results of different perturbation strategies. ‘w/o’ = ‘without’.

Configuration	mIoU (%)
w/o Speckle Noise	91.71 ± 2.48
w/o Low-pass Filtering	91.48 ± 2.46
w/o Phase Error	91.79 ± 2.49
**SSPNet (K = 100)**	**91.85 ± 2.44**

**Table 10:** Experimental results under different SNRs. ‘clean’ denotes the experimental results from Section 4.3, with metric statistics covering only these three target classes for consistency with the noisy experiments.

SNR (dB)	IoU per Category (%)			F1score per Category (%)			Overall Metrics (%)		
	Bkg.	Body	Panel	Bkg.	Body	Panel	OA	mIoU	mF1
−5	99.84 ± 0.04	85.92 ± 6.08	89.75 ± 2.46	99.92 ± 0.02	92.32 ± 3.60	94.55 ± 1.40	99.84 ± 0.03	91.84 ± 2.04	95.60 ± 1.20
0	99.85 ± 0.03	86.15 ± 5.97	90.44 ± 2.00	99.92 ± 0.01	92.46 ± 3.52	94.94 ± 1.14	99.85 ± 0.03	92.14 ± 2.02	95.77 ± 1.18
5	99.85 ± 0.02	86.20 ± 6.16	90.61 ± 1.63	99.93 ± 0.01	92.48 ± 3.63	95.04 ± 0.92	99.85 ± 0.02	92.22 ± 2.15	95.82 ± 1.26
10	99.85 ± 0.03	86.16 ± 6.13	90.67 ± 1.58	99.93 ± 0.01	92.46 ± 3.62	95.07 ± 0.90	99.85 ± 0.02	92.23 ± 2.12	95.82 ± 1.24
15	99.85 ± 0.03	86.22 ± 5.93	90.79 ± 1.68	99.93 ± 0.01	92.51 ± 3.49	95.14 ± 0.96	99.85 ± 0.03	92.29 ± 2.06	95.86 ± 1.20
20	99.85 ± 0.03	86.21 ± 5.83	90.89 ± 1.63	99.93 ± 0.01	92.50 ± 3.43	95.19 ± 0.93	99.85 ± 0.03	92.32 ± 1.96	95.87 ± 1.15
**clean**	**99.89 ± 0.03**	**91.46 ± 3.20**	**92.90 ± 1.93**	**99.95 ± 0.01**	**95.51 ± 1.76**	**96.29 ± 1.08**	**99.89 ± 0.02**	**94.75 ± 0.99**	**97.25 ± 0.54**

## Data Availability

The data that support the findings of this study are available from the corresponding author upon reasonable request.
